# User-generated content and influencer marketing involving e-cigarettes on social media: a scoping review and content analysis of YouTube and Instagram

**DOI:** 10.1186/s12889-023-15389-1

**Published:** 2023-03-20

**Authors:** Marissa J. Smith, Christina Buckton, Chris Patterson, Shona Hilton

**Affiliations:** MRC/CSO Social and Public Health Sciences Unit, University of Glasgow, Glasgow, UK

**Keywords:** Electronic cigarettes, e-cigarettes, Vaping, Social media

## Abstract

**Background:**

Evidence suggests that experimentation with e-cigarettes among young people is increasing. Social media is widely used by young people with user-generated content and influencer marketing particularly influential in promoting products. This paper documents a snapshot of online user-generated content and influencer marketing related to e-cigarettes on YouTube and Instagram.

**Methods:**

Scoping review of relevant e-cigarette-related content on two social media platforms popular with youths, YouTube and Instagram, between June and August 2021. Content analysis was undertaken to examine text, audio, and video content, recording age restrictions, health warnings, page characteristics, and post characteristics. Narrative post content was coded using a coding frame that was developed inductively in response to emergent categories.

**Results:**

Vaping was portrayed positively on social media; of the posts analysed, 86.5% (n = 90 of 104) of Instagram posts and 66.0% (n = 64 of 97) of YouTube videos. Warnings about age restrictions and health (e.g., nicotine addiction/toxicity) did not feature in the majority of posts; 43.3% (n = 42) of YouTube videos (n = 42) contained an age warning compared to 20.2% of Instagram posts (n = 21). While 25.8% (n = 25) of YouTube videos and 21.2% of Instagram (n = 22) posts contained a health warning.

**Conclusion:**

Of concern is the fact that the vast majority of YouTube and Instagram content about e-cigarettes promoted their use, and typically the content does not contain age and/or health warnings. These findings may highlight a priority for governmental policy to restrict the ability of marketers to reach youths with social media content promoting e-cigarettes.

**Supplementary Information:**

The online version contains supplementary material available at 10.1186/s12889-023-15389-1.

## Background

Electronic cigarettes (e-cigarettes, also known as Electronic Nicotine Delivery Systems or ENDS) are devices that heat a solution (e-liquid) to create an aerosol that can be inhaled through a mouthpiece and expelled similar to tobacco cigarette smoke [1, 2]. E-liquids are liquid solutions that are consumed in e-cigarettes, they are available in a variety of nicotine concentrations and often contain flavourings [3, 4]. In 2020, survey research indicates that over 15,000 different flavours were available on the market [5, 6]. Research has shown that the variety of available flavours is one of the top reasons for experimentation with e-cigarettes among youths, in addition to peer influence and curiosity [7–10]. Although e-cigarette use may reduce harm among adult smokers, research indicates that it could place adolescents at greater risk for adverse outcomes including other substance use and nicotine dependence [11, 12]. Due to sensitivities in the developing brain, adolescents are especially vulnerable to developing dependence following nicotine exposure [11, 13]. Research has identified a variety of chemical components in the cartridges, e-liquids, and aerosols of e-cigarettes [14, 15] such as nicotine, tobacco-specific nitrosamines, aldehydes, metals, volatile organic compounds, flavours, tobacco alkaloids, and drugs (such as amino-tadalafil and rimonabant) [16–18], though the long-term effects of these exposures among vapers remain unclear [19]. Nicotine, which most e-cigarettes contain, is a highly addictive chemical that has detrimental effects on brain cells and blood vessels and causes cardiovascular disease and brain diseases [20]. The long-term health effects associated with e-cigarette use are still unknown and as this evidence emerges, researchers will have a clearer understanding of the health risks and benefits associated with e-cigarette use.

The use of e-cigarettes among adults in England has remained stable since 2015; however, experimentation among young people has increased [21]. The legal age of vaping in the UK is 18. Despite differences in opinion within the public health community regarding the value of e-cigarettes in harm reduction for adults, there is broad consensus on the need to protect young people from this disruptive technology [22].

The e-cigarette market, which has traditionally been dominated by small companies, has experienced rapid growth and transition [23]. In 2019, the global value of the e-cigarette market was worth $11.73 billion and is expected to reach $21.4 billion by 2023 [24]. Major transnational tobacco companies (e.g., British American Tobacco and Philip Morris International) have entered the vaping industry by either acquiring e-cigarette companies and brands or developing their own products [25, 26]. These companies have benefited from large advertising and marketing budgets, which enable promotion across the internet and social media [27].

A range of regulatory approaches have been adopted in relation to the advertising of e-cigarettes, from being completely prohibited (e.g., Denmark and Australia) [33, 34] to being allowed as long as the advertisement adheres to certain regulations. Canada permits the advertising of e-cigarettes but prohibits lifestyle advertising, advertising that is appealing to youth [35]. The advertising of nicotine-containing e-cigarettes (unless licensed as medicines) in the UK is prohibited. Although regulations prohibits advertising in online media, social media content for e-cigarettes is permitted in “non-paid-for space online under the marketer’s control” providing that the content is “factual” rather than “promotional” [36]. Promotional content is deemed to include health claims, descriptive language, or significant imagery unrelated to the product, that goes beyond objective facts [36].

Social media platforms (including Instagram, Facebook, TikTok, YouTube, and Twitter) allow individuals and companies to create, share and interact with content including text, videos, photos, and links [37]. Social media companies can use social media to precisely target consumers’ specific demographic profiles, learn about who interacts with their content and provide real-time data on effectiveness, therefore it can be argued that it is a powerful marketing tool [37, 38]. Social media content can be generated by both influencers, individuals with a large following who are paid to promote bands and products online [38], and ‘regular’ people who may support a product or brand but without the formal endorsement of the company or any form of remuneration. Most adolescents use visual-based social media daily and are therefore susceptible to influence by both forms of content without necessarily differentiating between the two. Social media, therefore, has the capacity to influence young people’s understanding of products, including e-cigarettes [39]. E-cigarettes are promoted creatively through social media, with well-designed features including colours, flavour variations, incentives (such as price promotions and discount vouchers), and even celebrity endorsements [40]. Online marketing that leads to exposure to e-cigarette advertising, including where it is concealed as information or recommendations from peers, can increase the likelihood of vaping in individuals, including among younger people and non-smokers [41–43]. Instagram is one platform popular among teenagers and young adults through which e-cigarette brands (such as JUUL) have used visual advertising to market their products [44, 45]. In 2018, all corporate Instagram content related to JUUL posted before June 2017 was removed, unofficial community accounts (e.g., @juulnation and @juulnation) were removed and the official @juulvapour Instagram account was removed in response to growing public health concerns over JUUL’s popularity among youths [46, 47]. Previous studies have examined e-cigarette-related YouTube videos [48–53], focusing on characterising the portrayal of e-cigarettes and e-cigarette use and not recording other details (such as the prevalence of warning statements).

Given the near absence of regulation of e-cigarette marketing, and the potential influence of social media marketing on young people, it is important to understand of the nature, extent, and impact of e-cigarette marketing via social media to better understand whether policy interventions might be appropriate. This study, therefore, aimed to document the nature of online user-generated content and influencer marketing on social media platforms relating to e-cigarettes.

## Methods

### Selection of social media platforms

Based upon publicly-available data from Statista [54] we identified the six social media platforms most used by people aged 15–25 in the UK: YouTube (82% of respondents), Facebook (80%), WhatsApp (79%), Instagram (76%), Snapchat (57%), Twitter (44%). WhatsApp was excluded because of the impracticality and inappropriateness of studying the content of private, encrypted messages. Facebook was also excluded because sampling organically on Facebook is very challenging. There are many different ways in which content is organised, and the search options are not as powerful or transparent as YouTube. While Kong and colleagues [55] analysed marketing on brand-sponsored profiles, our focus on user-generated and influencer content suggests that more ‘grass-roots’ pages/groups might be relevant. You can search for Facebook groups or for specific pages (e.g., about vaping), but it is not possible to filter them by location or rank them by popularity. Ultimately, Facebook’s search is not transparent or flexible enough to provide robust search results of the type of data we were interested in (i.e., popular, impactful posts).

We decided to focus our analysis on YouTube and Instagram. As well as representing the two most-used sites with publicly shared content, they represent a spread of different content formats: predominantly video and predominantly images, respectively.

Between June and August 2021, we searched YouTube and Instagram for e-cigarette related videos and posts (using a UK IP address). When searching the social media platforms, we used a ‘clean’ (unused, logged out) web browser and created new accounts on platforms where required. These steps limited (but did not remove entirely) the ability for platforms to tailor search results based algorithmically on what is known about the user. All data collected were publicly available, meaning any person with an internet connection was able to view the data at the time it was retrieved.

#### YouTube

We conducted two separate searches on YouTube videos (in English) relating to e-cigarettes and vaping, see Additional file 1 for search details. Two researchers (CP and CB) independently analysed the fifty most-viewed videos in English. We anticipated that a larger sample would have been difficult to code reliably with the resources we had available. Total view count was used to sort the videos to collect a sample of e-cigarette-related videos that reached the largest audience, and therefore have the largest potential impact on public perceptions of e-cigarettes. Inclusion of fifty videos most likely captures items that are contained in the first couple of search pages generated by YouTube. Duplicate videos that appeared for multiple search terms were only counted and reviewed once. We acknowledge that our ability to be systematic and replicable was unavoidably limited by the bias introduced by the search algorithms, however, we were as systematic and transparent as possible within those limitations.

#### Instagram

In comparison to YouTube, search options on Instagram are very limited and the search field does not permit the use of complex search terms. In addition, Instagram only reveals nine of what it considers to be the ‘Top posts’ on the day of searching. To overcome this nine-post limit, we searched Instagram for multiple hashtags including: #ecig, #ecigarettes, #vape, #vaping, #vype, #vuse, #vapestick, #joytech, #vapourizer, #vaporizer, #vapelife, #juul, and #juulpods. A hashtag is a string of characters preceded by the symbol (#), which represents the core idea expressed in a social media post [56]. Hashtags also explain additional meanings, such as experience, and personal values, and portray social relationships [57].

### Coding and analysis

To analyse the videos and posts, two codebooks were developed. Two of the co-authors (MS and CB) took an inductive and iterative approach to coding the YouTube videos and Instagram posts and ensured that the codes were suited to the goals of the study.

For YouTube videos, reviewers recorded the number of views, likes, and dislikes, date posted, who posted the video (user-generated or organisation/company), what country the video was published/produced in, how many subscribers there were to the account that posted the video, if the video was sponsored, if any age warning was displayed (in the video description and/or in the video itself and/or if age verification was required), if any health warnings were displayed, if any promotions and or incentives for vaping products were displayed (in the video description and/or in the video itself). Finally, the overall depiction of e-cigarettes in the video was coded as positive (promoting products/use), negative (discouraging products/use), or neutral (open to viewer interpretation). MS and CB independently double-coded a random sample of 20 different videos and any disagreements were discussed and clarified, thus are able to provide intercoder reliability. YouTube restricts minors younger than 18 years from videos with inappropriate content, such as vulgar language, violence and disturbing imagery and portrayal of harmful or dangerous activities [58]. Viewers must register their date of birth on their Google account to access these videos. However, users not signed into an age-verified account could access all other videos that are not flagged as inappropriate. On instances where we were required to sign in to verify our age, the video was classified as having an age warning.

The inductive themes were constructed through a process of two researchers watching a subsample of videos independently, then meeting to discuss and compare emergent codes. Codes were refined to be operational with clear and concise definitions. See Additional file 2 for all coding categories.

For Instagram, we recorded the number of likes (a metric of user engagement [59, 60] and an important indicator of a post’s impact [61, 62]), date posted, who posted the image (user-generated or organisation/company), what country the post was published/produced in, if the image was sponsored, if an age warning was displayed (in the post description and/or in the post itself), if any health warnings were displayed, if any promotions and or incentives for vaping products were displayed (in the post description and/or in the post itself) and were any other accounts tagged in the post (e.g., vaping brands or manufactures). Finally, the overall depiction of e-cigarettes in the post was coded as positive (promoting products/use), negative (discouraging products/use), or neutral (open to viewer interpretation). MS and CB independently coded a random sample of 20 different post.

The posts were then categorised based on the coding frame developed for the analysis of the YouTube videos, supplemented by inductive coding. The inductive themes were constructed through a process of MS and CB independently coding a subsample of posts, then meeting to discuss and compare emergent codes. Codes were refined to be operational with clear and concise definitions. See Additional file 3 for all coding categories.

### Results

#### YouTube

Of the 100 YouTube videos identified, 97 were included in the study; three were excluded due to content being disabled. The videos ranged in length from 23 s to 18 min. Table 1 summarises descriptive features of the sample. The majority of videos (60.8%, n = 59 of 97) were posted in 2020. Of the 97 videos, 76.3% (n = 74) were user-generated and 23.7% (n = 23) were organisation/company videos. The majority of videos were produced in the US (76.3%, n = 74), with few produced in Europe (countries in the European Union) (3.1%, n = 3). The majority of videos (77.3%, n = 75) were not sponsored and of the 22.7% (n = 22) that were sponsored; the most common was public information sponsors (such as U.S. Food and Drug Administration and The Facts Now-Tobacco Free Florida campaign). Only 43.3% (n = 42) of videos contained an age warning (either in the video and/or required age verification) and health warnings were contained in 25.8% (n = 24) of videos. Health warnings included “this product can be harmful to your health”, “nicotine is an addictive chemical”. Some of the videos contained a combination of age and/or health warnings: 18.6% (n = 18) of videos contained an age warning and no health warning, 1.0% (n = 1) of videos contained a health warning and no age warning, 24.7% (n = 24) of videos contained an age warning and a health warning and 55.7% (n = 54) of videos contained no age warning and no health warning. In 65.9% (n = 64), the depiction of e-cigarettes was positive; 28.9% (n = 28) depicted e-cigarettes negatively and 5.2% (n = 5) were neutral. Few videos (2.1%, n = 2) contained or referred to any promotions or incentives for vaping products. The most common category of video was product review/information (45.3%, n = 44), with Autonomous Sensory Meridian Response (ASMR) featuring vaping (6.2%, n = 6), individual health warning (6.2%, n = 6) and news/TV segment (6.2%, n = 6) being least common. ASMR describes “the experience of tingling sensations in the crown of the head, in response to a range of audio-visual triggers such as whispering, tapping, and hand movements” [63, p.1].

Table 2 illustrates the details of the videos across all categories.

**Table 1:** Details of the 97 YouTube videos

Date posted	Number of videos (n = 97)
2020	59 (60.8%)
2021	38 (39.2%)
**Who posted**	
User-generated	74 (76.3%)
Organisation/company	23 (23.7%)
**Country posted in**	
Asia	1 (1%)
Canada	7 (7.2%)
Europe	3 (3.1%)
UK	74 (76.3%)
Unknown	3 (3.1%)
USA	9 (9.3%)
**Sponsorship**	
Yes	22 (22.7%)
No	75 (77.3%)
**Age warning**	
Yes	42 (43.3%)
No	55 (56.7%)
**Health warning**	
Yes	25 (25.8%)
No	72 (74.2%)
**Promotion or incentive**	
Yes	2 (2.1%)
No	95 (97.9%)
**Stance**	
Positive	64 (66.0%)
Negative	28 (28.9%)
Neutral	5 (5.1%)
**Categorisation**	
ASMR featuring vaping	6 (6.2%)
Individual health warning	6 (6.2%)
News/TV segment	6 (6.2%)
Product review/information	44 (45.4%)
Public health information	15 (15.5%)
Vape related behaviour	7 (7.2%)
Vape tricks/pranks/art	13 (13.4%)

**Table 2:** Details of the 97 YouTube videos based on categorisation

Category	Total (n = 97)*	Stance towards e-cigarettes**			Sponsored*	Age warning*	Health warning*	Incentive*
		Positive	Negative	Neutral				
ASMR featuring vaping	6 (6.2%)	6 (100%)	0 (0%)	0 (0%)	0 (0%)	4 (66.6%)	1 (16.6%)	0 (0%)
Individual health warning	6 (6.2%)	0 (0%)	6 (100%)	0 (0%)	0 (0%)	0 (0%)	0 (0%)	0 (0%)
News/TV segment	6 (6.2%)	0 (0%)	6 (100%)	0 (0%)	1 (16.7%)	0 (0%)	0 (0%)	0 (0%)
Product review/information	44 (45.4%)	43 (97.7%)	0 (0%)	1 (2.3%)	5 (11.4%)	33 (75%)	22 (50%)	1 (2.3%)
Public health information	15 (15.5%)	0 (0%)	15 (100%)	0 (0%)	15 (100%)	0 (0%)	0 (0%)	0 (0%)
Vape related behaviour	7 (7.2%)	5 (71.4%)	0 (0%)	2 (28.6%)	0 (0%)	1 (14.3%)	2 (28.6%)	0 (0%)
Vape tricks/pranks/art	13 (13.4%)	10 (76.9%)	1 (7.7%)	2 (15.4%)	1 (7.7%)	4 (30.8%)	0 (0%)	1 (7.7%)

*Percentages are of column total

**Percentages are within stance

The six videos categorised as ASMR portrayed e-cigarettes positively. Four of the six videos included an age warning, and one contained a health warning. In contrast to individual health warning and public health information categories, where e-cigarettes were portrayed negatively, the videos in these two categories did not include age and/or health warnings. Product review/information, which was the most common category, mostly depicted a positive stance on e-cigarettes and one video included a promotion/incentive to buy vaping products. However, it is noteworthy that the videos in this category often contained age and/or health warnings. Similarly, in the vape-related behaviour and vape tricks/pranks/art categories, where vaping was portrayed positively, videos were found to contain age and/or health warnings.

#### Instagram

Of the 117 Instagram posts retrieved, 104 were included in the study; six were excluded due to content being disabled and seven did not include vaping products or related activity. Table 3 shows further details of the 104 posts. The year of posting ranged from 2014 to 2021 (median = 2018, IQR = 2015–2020). Overall, 65.4% (n = 68 of 104 posts) were user-generated and 34.6% (n = 36) were organisation/company posts. A quarter of the posts were produced/posted in Europe (25.0%, n = 26), with few posts originating in Africa (1.9%, n = 2), Asia (1.9%, n = 2), Australia (1.9%, n = 2), Jordan (1.9%, n = 2) and Mexico (1.9%, n = 2). Only 20.2% (n = 21) contained an age warning (either in the post and/or post description) and health warnings were contained in 21.2% (n = 22) of posts. Some posts contained a combination of age and/or health warnings: 2.9% (n = 3) of the posts contained an age warning and no health warning, 3.8% (n = 4) of the posts contained a health warning and no age warning, 17.3% (n = 18) of the posts contained an age warning and a health warning and 76.0% (n = 79) of the posts contained no age warning and no health warning, Few posts (15.4%, n = 16) included a promotion or incentive to purchase vaping products. In 86.5% (n = 90), the depiction of e-cigarettes was positive; 1.0% (n = 1) depicted e-cigarettes negatively and 12.5% (n = 13) were neutral. The most common category of video was product review/information (70.2%, n = 73), with individual health warning (1.0%, n = 1) and public health information (1.0%, n = 1) being least common. Table 4 illustrates the details of the posts across all categories.

**Table 3:** Details of the 104 Instagram posts

Date posted	Number of posts (n = 104)
2014–2018	6 (5.8%)
2019–2021	98 (94.2%)
**Who posted**	
User-generated	68 (65.4%)
Organisation/company	36 (34.6%)
**Country posted in**	
Africa	2 (1.9%)
Asia	3 (2.9%)
Australia	2 (1.9%)
Canada	4 (3.8%)
Europe	26 (25.0%)
Jordan	2 (1.9%)
Mexico	2 (1.9%)
UK	24 (23.1%)
USA	23 (22.1%)
Unknown	12 (11.5%)
**Sponsorship**	
Yes	0 (0%)
No	104 (100%)
**Age warning**	
Yes	21 (20.2%)
No	83 (79.8%)
**Health warning**	
Yes	22 (21.2%)
No	82 (78.8%)
**Promotion or incentive**	
Yes	16 (15.4%)
No	88 (84.6%)
Stance	
Positive	90 (86.5%)
Negative	1 (1.0%)
Neutral	13 (12.5%)
**Categorisation**	
Competition or giveaway	2 (1.9%)
Individual health warning	1 (1.0%)
Meme	2 (1.9%)
Other	3 (2.8%)
Personal photograph	12 (11.5%)
Product review/information	73 (70.2%)
Public health information	1 (1.0%)
Vape tricks/pranks/art	10 (9.6%)

**Table 4:** Details of the 104 Instagram posts based on categorisation

Category	Total (n = 104)*	Stance towards e-cigarettes**			Age warning*	Health warning*	Incentive*
		Positive	Negative	Neutral			
Competition/giveaway	2 (6.2%)	2 (100%)	0 (0%)	0 (0%)	0 (0%)	0 (0%)	2 (100%)
Individual health warning	1 (6.2%)	0 (0%)	1 (100%)	0 (0%)	0 (0%)	0 (0%)	0 (0%)
Meme	2 (6.2%)	2 (100%)	0 (0%)	0 (0%)	0 (0%)	0 (0%)	0 (0%)
Other	3 (45.4%)	0 (0%)	0 (0%)	3 (100%)	0 (0%)	0 (0%)	0 (0%)
Personal photograph	12 (7.2%)	8 (66.7%)	0 (0%)	4 (33.3%)	0 (0%)	0 (0%)	0 (0%)
Product review/information	73 (15.5%)	72 (98.6%)	0 (0%)	1 (1.4%)	21 (28.8%)	22 (30.1%)	14 (19.2%)
Public health information	1 (13.4%)	0 (0%)	0 (0%)	1 (100%)	0 (0%)	0 (0%)	0 (0%)
Vape tricks/pranks/art	10 (9.6%)	6 (60.0%)	0 (0%)	4 (40.0%)	0 (0%)	0 (0%)	0 (0%)

*Percentages are of column total

**Percentages are within stance

The two posts categorised as competition/giveaway, unsurprisingly portrayed a positive stance and included an incentive/promotion to buy vaping products. The one video categorised as individual health warning was the only post to depict a negative stance but did not include a health warning. Product review/information, which was the most common category, predominately depicted a positive stance on e-cigarettes and several posts (n = 14) included a promotion/incentive to buy vaping products. However, it is noteworthy that the posts in this category often contained age and/or health warnings and were the only category to include age and/or health warnings.

#### Comparison across two platforms

Comparing the results across the two social media platforms identified several similarities and differences. In the YouTube search, the upload date was filtered to 2021, however, 2020 videos were included in the sample. Whereas on Instagram, we examined the ‘top posts’ relating to each hashtag, meaning the date was unrestricted and resulted in a wider range (median = 2018, IQR = 2015–2020). Unlike YouTube, where 22.7% (n = 22) of the videos were sponsored, none of the Instagram posts analysed included sponsorship. Comparison of the presence of age warnings highlighted that a higher percentage of YouTube videos (43.3%, n = 42) contained an age warning compared to Instagram posts (20.2%, n = 21). Concerning health warnings were similar across the two platforms: YouTube (25.8%, n = 25) and Instagram (21.2%, n = 22). In relation to stance, selected Instagram posts predominately portrayed a positive stance towards vaping (86.5%, n = 90). Selected YouTube videos mostly portrayed a positive stance towards vaping (66.0%, n = 64), while 28.9% (n = 28) depicted a negative stance.

Across both YouTube and Instagram, the most common category was product review/information, and the depiction of e-cigarettes was predominately positive. In contrast to other categories, this category, across both platforms, often contained age and/or health warnings. Vape tricks/pranks/art was a common theme across both platforms, and the depiction of e-cigarettes was positive in this category on both platforms. YouTube videos included in this category often contained an age warning, but posts on Instagram did not. Individual health warning was a less common category on both platforms, and the videos/posts in this category portrayed a negative stance towards e-cigarettes.

## Discussion

Our study examined the content and characteristics of vaping videos on YouTube and posts on Instagram. In doing so, we offered key insights into the prevalence of vaping-related content on social media platforms. The dominant presence of reviews of vaping products and the lack of age and health warnings highlights that social media can expose youths to an array of products meant for adult use only. Such techniques have also been used historically by the tobacco industry to advertise combustible cigarettes [64–66]. We found that the vast majority of information on YouTube and Instagram about vaping promotes products/use and depicts the use of e-cigarettes as socially acceptable. This can have implications for youths who may be susceptible to such advertising. Although there are methods to deter youths from viewing the content (age and/or health warnings), we found a large proportion of the videos and posts did not use such methods (see Tables 2 and 4 for more details). Several videos and posts also provided incentives or promotions to buy vaping products, which is concerning because past research has found that some websites do not verify age when selling tobacco products online [67, 68]. Considering that most youth access multiple social media platforms multiple times per day [69, 70] and that exposure to this marketing is related to use [71, 72], this combination of findings indicates a significant public health concern.

While several social media platforms offer functions to page owners to restrict the ages of viewers (Additional file 4), no platforms require the use of age restrictions for all tobacco-related content, and vaping companies/organisations’ pages may not voluntarily elect to employ these restrictions [73]. As highlighted by the results from our study, the majority of YouTube videos and Instagram posts are not restricted and/or contain age and/or health warnings. Based on our analysis we cannot for certain state that youths are extensively exposed to vaping content but we can state that should youths actively access this content or be exposed to this indirectly, a large portion of vaping content we found promotes e-cigarette use, and typically the content does not contain age and/or health warnings, which is concerning.

Previous studies that have examined e-cigarette-related YouTube videos [48–53] have focused on characterising the portrayal of e-cigarettes and e-cigarette use and not the prevalence of warning statements. While this was the primary focus of our research, we recorded other relevant details including, age and health warnings. It is important that the prevalence of warning statements on YouTube, and other social media platforms, be documented to inform potential policy action. Previous research [74–76] which examined tobacco-related videos, found that the majority of videos promoted tobacco use and as highlighted by our study, we are seeing a similar depiction of vaping products on YouTube. Similar to previous studies [77–80], we found that the vast majority of the Instagram posts we examined depicted positive attitudes towards vaping, while negative characterisations were mostly absent. Consequently, there is a risk that youths will be exposed to, and possibly engage with, content that promotes vaping while staying uninformed about the negative aspects, including potential health harms. In line with previous studies [77, 79, 80], our study found that an overwhelming amount of content related to vaping on YouTube and Instagram promoted e-cigarette use, while the negative facet was mostly absent. Given the findings presented by our study, social media platforms should consider implementing more robust measures, such as age restrictions and portraying the negative aspects of vaping, to ensure the prevention of vaping-related content targeted at underage users. Policymakers should require social media platforms to build more robust measures to protect youths and to restrict the ability of marketers to reach youths with social media content promoting e-cigarettes. Policymakers may also consider implementing campaigns and/or online resources to educate parents on vaping, the health risks of vaping and/or nicotine addiction and its appeal to youths. Furthermore, we should urge parents to talk to their children about vaping, the health risks associated with vaping the content they may be exposed to or come across while on social media. Parents may also consider implementing parental controls on the type of content youths can access, particularly for younger children.

Our study has several strengths. We identified vaping-related content across two social media platforms popular with young people, and this represents an important step towards understanding what vaping-related content may reach various audiences, and young people in particular. We carried out an in-depth investigation of the sampled content from both platforms, with independent validation of coding to maximise reliability. However, several limitations should be noted. First, the findings from our study are time-limited, as this study analysed a cross-section of social media from June-August 2021. The videos and posts we analysed represented snapshots of a portion of the relevant content on Instagram and YouTube on the days that the searches were conducted. Search algorithms may return different types of results depending on the time and day when are conducted. For example, our searches were conducted on a weekday, so it is useful to acknowledge that the results may have been different on a weekend day, depending on the search algorithms. Similarly, social media users may post different types of content on different days. Second, our content analysis was based on 97 YouTube videos and 104 Instagram posts retrieved through keyword searches, which might not represent a comprehensive snapshot of e-cigarette-related content on those platforms, although our focus on ‘Top Posts’ and the most-viewed videos means that our sample focuses on the content with the broadest reach and high potential for influence. Third, we limited our search on YouTube to English-language videos, limiting the generalisability of the results to non-English-speaking populations. Fourth, we did not examine the content of user comments posted beneath videos and images, but we acknowledge that those comments represent part of the content that may be read by users. Instead, we focused on the content of the video or post and the caption (if provided) of the video or post. Fifth, although we adhered to rigorous and standard methods for qualitative analysis, this approach is not without some level of subjectivity and thus other researchers may code, define, and interpret data differently. Our methodology is consistent with trends within content analysis practice towards a more interpretative approach, that acknowledges the limits of objectivity in coding media content [81]. We believe that the categories capture the nature of the social media posts and could provide a framework to be used in future studies. Finally, although we did record the country where the video/post was produced, this study did not consider differences in geographic locations as YouTube and Instagram are international platforms. It is possible that the country of origin where the search was conducted (the UK) could influence the search results produced. However, different geographical locations and their impacts in different countries could be pursued in future studies.

Future research could use machine-learning techniques to examine vaping-related content on social media for a large-scale analysis. This would allow for an examination of one platform or for comparison across platforms, similar to our study, but on a large scale. In addition, future research could explore the broader influence of social media marketing on youth e-cigarette use by measuring the attention that young people pay to vaping advertising, or by conducting focus groups among diverse groups of youths to gauge their perceptions of and engagement with this content.

## Conclusion

The use of social media platforms for advertising and promoting vaping and vaping products raises concerns that youths may be attracted to products that are designed for adult use only. The results of this study highlight that a large portion of vaping content shared on YouTube and Instagram promotes e-cigarette use, and typically the content does not contain age and/or health warnings. Most adolescents use social media daily and depiction of products on social media represents a key influence on young people’s understandings of products. These findings may highlight a priority for governmental policy to restrict the ability of marketers to reach youths with social media content promoting e-cigarettes.

## Electronic supplementary material

Below is the link to the electronic supplementary material.

Supplementary Material 1

Supplementary Material 2

Supplementary Material 3

Supplementary Material 4

## Data Availability

The datasets used and/or analysed during the current study are available from the corresponding author on reasonable request.

## List of abbreviations

ASMR: Autonomous Sensory Meridian Response
e-cigarettes: Electronic cigarettes
