# Supplementary material for: User-generated content and influencer marketing involving e-cigarettes on social media: a scoping review and content analysis of YouTube and Instagram
Source: BMC Public Health. 2023 Mar 20;23:530. doi: 10.1186/s12889-023-15389-1 (PMC10029293; doi:10.1186/s12889-023-15389-1)
Supplement: Supplementary file 1 — Supplementary Material 1 [file 12889_2023_15389_MOESM1_ESM.docx]

**Additional file 1 Search details of the two YouTube searches**

The search terms used in Search 1 focused on e-cigarettes and vaping. Based upon a team discussion, it was decided to include other e-cigarette and vaping related search terms(including reference to specific brands) as it would enable us to capture more e-cigarette content on YouTube.

| **Search** | **Search String** | **Filters** |
| --- | --- | --- |
| 1 | “electronic cigarette" OR "e-cigarette" OR "ecigarette" OR "vape" OR "vaping" | Upload date: 2021  Type: video  Sort by: view count |
| 2 | “ecigarette" OR "e-cigarette" OR "vape" OR "vaping") AND ("Blu" OR “Vype” OR “Vuse” OR “Juul” OR “E-Lites” OR “IQOS” OR “Vapestick” OR “Joyetech” OR “OK eCig” OR “Vapouriz” OR “Liberty Flights” OR “MultiCig” | Upload date: 2021  Type: video  Sort by: view count |
