# Supplementary material for: User-generated content and influencer marketing involving e-cigarettes on social media: a scoping review and content analysis of YouTube and Instagram
Source: BMC Public Health. 2023 Mar 20;23:530. doi: 10.1186/s12889-023-15389-1 (PMC10029293; doi:10.1186/s12889-023-15389-1)
Supplement: Supplementary file 2 — Supplementary Material 2 [file 12889_2023_15389_MOESM2_ESM.docx]

**Additional file 2: Full coding framework for the analysis of the YouTube videos**

| **Category** | **Description** |
| --- | --- |
| Autonomous Sensory Meridian Response (ASMR)^[[1]](#footnote-2)^ featuring vaping | Shows ASMR using or featuring vaping products |
| Individual health warning | Portrays use as providing health benefits, reducing harm, or not being harmful to one’s health |
| News/TV segment | News or TV broadcast featuring or discussing vaping |
| Product review/information | Review of a vape product and/or providing information of vape products. This could feature one vape produce or a comparison to other vape products. |
| Public health information | Shows or discusses public health information (such as health effects) concerning vape products |
| Vape related behaviour | Shows or discusses vape related behaviour (e.g., how to use a vape and how to assemble products) |
| Vape tricks/pranks/art | Video contains individual or a group performing tricks (e.g., smoke rings), pranks and/or art with vape products. |

1. ASMR describes “the experience of tingling sensations in the crown of the head, in response to a range of audio-visual triggers such as whispering, tapping, and hand movements.” (56, p.1) [↑](#footnote-ref-2)
