# Supplementary material for: User-generated content and influencer marketing involving e-cigarettes on social media: a scoping review and content analysis of YouTube and Instagram
Source: BMC Public Health. 2023 Mar 20;23:530. doi: 10.1186/s12889-023-15389-1 (PMC10029293; doi:10.1186/s12889-023-15389-1)
Supplement: Supplementary file 3 — Supplementary Material 3 [file 12889_2023_15389_MOESM3_ESM.docx]

**Additional file 3: Full coding framework for the analysis of the Instagram posts**

| **Category** | **Description** |
| --- | --- |
| Competition or giveaway | Post describes or promotes a competition or giveaway of vape products |
| Individual health warning | Portrays use as providing health benefits, reducing harm, or not being harmful to one’s health |
| Meme | An image, video, piece of text, etc., typically humorous in nature, that is copied and spread rapidly by internet users |
| Other | Where posts did not fall within one of the other predefined categories |
| Personal photograph | Post is a personal photograph posted by the user which includes a vaping product |
| Product review/information | Review of a vape product and/or providing information on vape products. This could feature one vape product or a comparison to other vape products. |
| Public health information | Shows or discusses public health information (such as health effects) concerning vape products |
| Vape tricks/pranks/art | Post contains individual or a group performing tricks (e.g., smoke rings), pranks, and or art with vape products |
