# Supplementary material for: User-generated content and influencer marketing involving e-cigarettes on social media: a scoping review and content analysis of YouTube and Instagram
Source: BMC Public Health. 2023 Mar 20;23:530. doi: 10.1186/s12889-023-15389-1 (PMC10029293; doi:10.1186/s12889-023-15389-1)
Supplement: Supplementary file 4 — Supplementary Material 4 [file 12889_2023_15389_MOESM4_ESM.docx]

**Additional file 4: Social media user information and tobacco-related policies**

| **Platform** | **Can restrict the visibility of posts by age?** | **Policies related to tobacco advertising** |
| --- | --- | --- |
| Facebook | Yes | Ads must not promote the sale or use of tobacco products and related paraphernalia. Advertisements must not promote electronic cigarettes, vaporisers, or any other products that simulate smoking. Blogs or groups connecting people with tobacco-related interests are permitted, as long as the service does not lead to the sale of tobacco or tobacco-related products. Anti-smoking campaigns and e-books, counselling services for smoking addiction and smoking rehabilitation programs or facilities are permitted [1]. |
| Instagram | Yes (by restricting all content on account) | Advertising follows the same regulations as Facebook. |
| Pinterest | No | Pinterest doesn’t allow the advertising of tobacco products including but not limited to cigarettes, e-cigarettes, vapes or vape cartridges, chewing tobacco, paraphernalia (i.e., pipes, rolling papers, vapes, vape cartridges, etc.), nicotine products (e.g., nicotine gum or patches) and any brands associated with the above products. Pinterest also does not allow the advertising of hookahs, hookah bars, cigars, or cigar bars, essential oil inhalers, or related products [2]. |
| Snapchat | No | Under inappropriate content for ads, it says; cigarettes (including e-cigarettes), cigars, smokeless tobacco, and other tobacco products [3]. |
| Tumblr | No | Tumblr does not allow the promotion of cigarette, cigar, or tobacco products and/or smoking in our advertising. This policy does not apply to characters or actors smoking, content related to smoking cessation and news and current events related to smoking [4]. |
| Twitter | Yes (but must restrict content to followers and require the follower to be of age) | Twitter prohibits the promotion of tobacco products, accessories, and brands globally. This includes tobacco of any kind, including chewing tobacco and imitations, all cigarettes, including alternatives that imitate the act of smoking, cigars, tobacco pipes, rolling papers, and filters, cigar bars and hookah lounges, tobacco manufacturers and events sponsored by tobacco manufacturers [5]. This applies to Twitter’s paid advertising, not to tweets |
| YouTube | Yes | Content that promotes tobacco and tobacco-related products is not suitable for advertising.  The following types of content are prohibited, videos of minors using vaporisers, e-cigarettes, tobacco or marijuana [6]. |

**References**

1. Facebook. Tobacco and related products 2022 [updated N.D. Available from: <https://www.facebook.com/policies/ads/prohibited_content/tobacco>.

2. Pinterest. Advertising Guidelines 2022 [updated N.D. Available from: [https://policy.pinterest.com/en/advertising-guidelines#:~:text=We%20don't%20allow%20the%20advertising%20of%20tobacco%20products%20including,Vapes%20or%20vape%20cartridges](https://policy.pinterest.com/en/advertising-guidelines#:~:text=We%20don't%20allow%20the%20advertising%20of%20tobacco%20products%20including,Vapes%20or%20vape%20cartridges).

3. Snap Inc. Snap Advertising Policies 2022 [updated N.D. Available from: <https://snap.com/en-GB/ad-policies>.

4. Tumblr Inc. Tumblr Global Advertising Policy 2022 [updated 19 January 2022. Available from: <https://www.tumblr.com/policy/en/global-advertising>.

5. Twitter Inc. Tobacco and tobacco accessories 2022 [updated N.D. Available from: <https://business.twitter.com/en/help/ads-policies/ads-content-policies/tobacco-and-tobacco-accessories.html>.

6. YouTube. Harmful or dangerous content policies 2022 [updated N.D. Available from: [https://support.google.com/youtube/answer/2801964?hl=en-GB#zippy=%2Cage-restricted-content%2Cdrug-use%2Cviolent-events-or-instructions-to-harm%2Cdangerous-or-threatening-pranks%2Cextremely-dangerous-challenges](https://support.google.com/youtube/answer/2801964?hl=en-GB#zippy=%2Cage-restricted-content%2Cdrug-use%2Cviolent-events-or-instructions-to-harm%2Cdangerous-or-threatening-pranks%2Cextremely-dangerous-challenges)
